# Editorial: Databases and nutrition, volume III

**DOI:** 10.3389/fnut.2025.1675443

**Published:** 2025-10-23

**Authors:** Alessandra Durazzo, Igor Pravst, Massimo Lucarini

**Affiliations:** ^1^CREA-Research Centre for Food and Nutrition, Rome, Italy; ^2^Institute of Nutrition, Ljubljana, Slovenia

**Keywords:** food data, food groups, nutrients, natural substances, dietary supplements, classification, categorization, food composition database

## Introduction

Public health nutrition is the promotion of the nutrition-related health of populations. Food composition databases have an essential role in the assessment, analysis, and action phases of public health nutrition. The food composition database provides comprehensive information on the energy content, various nutrients, and other bioactive constituents in food products obtained from agriculture, fisheries, and livestock. The country-specific food composition databases are developed to include the composition data of foods consumed by the population and represent essential tools for assessing national nutritional status, thus being critical to advance nutritional research and policy. The management of food composition programmes includes the maintenance and continuous updating of food composition information, as this is a useful tool for estimating nutrient intake at the national, regional, and/or certain population levels.

Accurate, country-specific food composition databases that reflect the national food supply are essential for estimating nutrient intake and conducting reliable dietary assessments, thereby serving as a key tool for evaluating and monitoring diets. Indeed, food composition databases are utilized to meet the supply and demand of agricultural products, assess the quality of exported products in international trade, public health campaigns, nutrition programmes and strategies, and boost innovations in the food industry.

Food composition databases provide reliable data on nutrient composition and bioactive profiles, supporting diverse applications such as clinical nutrition, epidemiological research, health surveys, diet therapy and planning, dietary guidelines, nutrition policies, food development, nutrition recommendations, nutrition education, and food labeling regulations. Therefore, food composition databases are fundamental for a broad user base, i.e., researchers, dietitians, clinical dietitians, and other health professionals, government policymakers, consumers, marketing professionals, and other policymakers. These databases are therefore also used in a wide variety of organizations—from academia to various industries, including food businesses, IT providers, and governments.

The integration and harmonization of food composition data and modern omics technologies is an ongoing challenge. Beyond the macro- and micronutrient information provided by national databases, resources for food composition data are increasingly focused on high-resolution analyses aimed at capturing the full spectrum of small, potentially bioactive molecules present in foods. The availability of standardized, harmonized, and integrated large-scale food composition data and mass spectra resources will be fundamental for future directions in the perspective of data integration and interoperability. Quality control of analytical procedures is a key element for the accuracy, precision, and reliability of data for inclusion in food composition databases.

Safe food represents a key aspect of food security, and, consequently, food traceability along the supply chain represents a fundamental component. Data traceability starts from data collection and continues through to analysis results; its role is to ensure data reproducibility along the food chain, from raw material production to transportation to logistics. The analytical data and the development of a food safety assessment system produce useful information and represent key elements to obtain an effective traceability system and guarantee efficiency in the management of the entire supply chain. Emerging technologies such as cloud computing, digital platforms, mobile tools, and artificial intelligence offer new opportunities to build smart food traceability systems that integrate across the agri-food supply chain. These systems can monitor food supply and population-level dietary data, thereby improving data quality and safety while supporting the development of integrated food data infrastructures. Particularly, the use of artificial intelligence is currently emerging as a key part of the management of food composition databases.

There is a need to improve the international harmonization of food composition databases to meet expectations for international research and comparisons. The classification and harmonization of foods are essential to the development of connectable database systems. The growing availability of standardized data facilitates integration across sources, as future analyses increasingly rely on data harmonization and interoperability. A key current challenge is linking environmental and food composition databases, connecting nutritional and environmental entries in order to identify more sustainable food options. Furthermore, there is a need for additional data regarding food waste and by-products and, consequently, for databases to include information on chemical composition, origin, and quantities of by-products from the agri-food sector.

The availability of branded food databases also brings new opportunities and challenges. By providing detailed and up-to-date nutritional information specific to branded products, these databases improve the reliability of data for applications such as nutrient intake assessment and food reformulation monitoring.

In this context, the present Special Section, *Databases and nutrition, volume III*, brings together nine contributions that address these themes from different perspectives. Concerning the development of automatic procedures in database management, the study of Westenbrink et al. addressed the development of an automated approach to identify fortified foods in the Dutch branded food database LEDA (short for LEvensmiddelenDAtabank). An automated procedure, based on a stepwise approach conforming to European food labeling legislation, using a list of rules and search terms, was developed and resulted in the identification of 1,817 foods, fortified with one or more of the selected nutrients in the LEDA dataset (0.94%; Westenbrink et al.).

The study of Bardon et al. described the development and evaluation of the FNS-Cloud data quality assessment tool for dietary intake datasets.

The study of Valenčič et al. presented NutriBase, a novel database and knowledge management system designed to advance the science of food composition through improvements in harmonization, data quality, reduction of missing data, and interoperability.

Regarding uses and applications of databases, the study of Fazzino et al. quantified the prevalence of hyper-palatable food (HPF) in the Italian food system and compared the hyper-palatability of similar foods across Italy and the United States (US), which has wide HPF saturation: HPF comprise less than one third of the Italian food system, indicating the Italian food system may confer protection from HPF exposure. Findings also revealed key differences in HPF products between Italy and the US, with HPF from Italy tending to have lower palatability-inducing nutrients and higher satiety-promoting nutrients relative to comparable US products (Fazzino et al.). Moreover, authors highlighted that food companies in Italy and the US should consider reducing the sodium, refined carbohydrates, and fat in salty snacks, frozen pizzas, industrial breads, and protein/cereal bars to reduce the hyperpalatability of these commonly consumed foods in Italy and the US.

Wang X. et al. investigated the association between plain water intake (PWI) and the risk of osteoporosis among middle-aged and elderly people in the United States by a cross-sectional study: results suggested that among middle-aged and elderly people, a greater PWI was connected with a moderately lower osteoporosis risk. The study of Wang Y. et al. is focused on the application of food composition data; their work was focused on the exploration of the links between consumption of Sugar-sweetened beverages (SSB) and specific health-related outcomes and lifestyle parameters.

Kraemer et al. have discussed methodological evolution and challenges of in-store census methods for assessing the composition of branded foods, and they characterized a Brazilian food label database.

Terro et al. present the IsoFoodTrack database—a comprehensive, scalable, and flexible platform designed to manage isotopic and elemental composition data for a wide range of food commodities. Brinkley et al. conducted an integrative review of 35 data attributes across 101 FCDBs from 110 countries, highlighting emerging opportunities and recommendations.

Contributions in Volume III of Databases and Nutrition showcase cutting-edge efforts to develop and update comprehensive and dedicated food databases, emphasizing rigorous standardization, harmonization, and interoperability across data sources—from analytical measurements to literature-derived values, labeling, and calculated data. The adoption of robust quality evaluation indices, consistent food description systems, and semi-automated matching and alignment procedures reflects the growing implementation of nutritional data infrastructures. These resources serve not only to support food composition research but also to underpin interdisciplinary applications spanning health, environmental science, policy, and beyond.

